# Delusion of Pregnancy: A Case Revisited

**DOI:** 10.3233/BEN-120324

**Published:** 2013

**Authors:** A. J. Larner

**Affiliations:** Cognitive Function Clinic, Walton Centre for Neurology and Neurosurgery, Lower Lane, Fazakerley, Liverpool, L9 7LJUK

**Keywords:** Delusion of pregnancy, frontotemporal dementia with motor neurone disease, C9ORF72 mutation

## Abstract

A patient with delusion of pregnancy as an early feature of frontotemporal dementia with motor neurone disease (FTD/MND) who was reported some years ago was posthumously found to harbor the C9ORF72 hexanucleotide repeat expansion, now known to be the most common genetic cause of FTD/MND. Some series have found psychosis to be common in FTD associated with this mutation, so this test should be considered in individuals with clinical features of FTD and MND and/or with a family history of either disorder who present with bizarre delusions.

## 1. Introduction

A patient with delusion of pregnancy in the context of frontotemporal dementia with motor neurone disease was reported in a previous issue of the Journal [1]. Further information on the aetiology of this case has now become available.

## 2. Case report

A 42 year-old woman presented with change in personality with prominent delusion of pregnancy. She was found to have clinical, neuropsychological, neuroradiological and neurophysiological evidence of frontotemporal dementia with motor neurone disease (FTD/MND). The patient's father was reported to have died of MND in his 60 s, prompting the suspicion of an inherited disorder, but at the time of investigation the only widely available test for a genetic mutation deterministic for FTD was for the tau gene (*MAPT*), which proved to be negative in this patient [1]. She subsequently died aged 44 from complications of her disease, some 18 months after diagnosis, consistent with the poor prognosis of FTD/MND [2]. Postmortem was not undertaken.

The discovery of a hexanucleotide (GGGGCC) repeat expansion in the C9ORF72 gene on chromosome 9p as deterministic for cases of inherited and sporadic FTD and MND, and particularly in families with both FTD and MND, was first reported in November 2011 [3,4]. These and subsequent studies [5–11] have suggested that this mutation may be responsible for around 25% of inherited FTD and 5% of sporadic FTD, and present in even higher frequencies in families with both FTD and MND.

Accordingly, a posthumous examination of the patient's stored DNA was undertaken. The abnormal C9ORF72 hexanucleotide repeat expansion was detected, confirming the previous clinical diagnosis and establishing its genetic aetiology.

## 3. Discussion

Some groups have noted that the presence of psychosis in FTD greatly increases the odds of finding the C9ORF72 mutation. For example, in a cohort from the United Kingdom [5] it was noted that 38% of mutation carriers presented with florid psychotic symptoms, for which initial psychiatric diagnoses of delusional psychosis, somatoform psychosis, and para-

ISSN 0953-4180/13/$27.50 © 2013 – IOS Press and the authors. All rights reserved noid schizophrenia had been made. An additional 28% had paranoid, delusional and irrational thinking. Delusions were much more common than hallucinations. In an Australian cohort of FTD patients the prevalence of psychotic features was significantly higher in those with C9ORF72 expansions (56% vs 14%) [11]. C9ORF72 expansions have not been found in patients diagnosed with schizophrenia [12].

Delusions which may be dramatic and bizarre but transient may sometimes occur in FTD/MND. Many patients with FTD/MND are seen by psychiatrists prior to diagnosis [13]. It will be of interest to learn if other patients with FTD/MND presenting with delusions carry the C9ORF72 mutation. Certainly consideration of genetic testing for this expansion would be appropriate in individuals with clinical features of FTD and MND and/or with a family history of either disorder who present with bizarre delusions.
